# The relation of lactate level and carbon dioxide pressure discrepancies between transcutaneous and arterial measurements

**DOI:** 10.5578/tt.202402920

**Published:** 2024-06-12

**Authors:** Aslıhan GÜRÜN KAYA, Şeyda Nur ÖZPINAR, Miraç ÖZ, Serhat EROL, Fatma ARSLAN, Aydın ÇİLEDAĞ, Akın KAYA

**Affiliations:** 1 Department of Chest Disease, Ankara University Faculty of Medicine, Ankara, Türkiye

## Abstract

**ABSTRACT**

**
The relation of lactate level and carbon dioxide pressure discrepancies between transcutaneous and arterial measurements
**

**Introduction:** *Partial carbondioxide pressure of the arterial blood (PaCO2) is used to evaluate alveolar ventilation. Transcutaneous carbon dioxide pressure (TcCO2) monitoring has been developed as a non-invasive (NIV) alternative to arterial blood gas analysis (ABG). Studies have shown that decreased tissue perfusion leads to increased carbondioxide (CO2). The use of transcutaneous capnometry may be unreliable in patients with perfusion abnormalities. In this study, we aimed to evaluate the relation between TcCO2-PaCO2 and lactate level which is recognized as a marker of hypoperfusion.
*

**Materials and Methods:** *In this prospective cohort study in critical care patients with hypercapnic respiratory failure (PaCO2 ≥45 mmHg) who received NIV between April 2019 and January 2020 in the intensive care unit were enrolled in the study. Patients’ simultaneously measured TcCO2 and PaCO2 values of hypercapnic patients were recorded. Each paired measure- ment was categorized into two groups; normal lactate (<2 mmol/L) and increased lactate (≥2 mmol/L).
*

**Results:** *A total of 116 paired TcCO2 and PaCO2 measurements of 29 patients were recorded. Bland-Altman analysis showed the mean bias between the TcCO2 and PaCO2 and 95% limits of agreement (LOA) in all measurements (1.75 mmHg 95% LOA -3.67 to 7.17); in the normal lactate group (0.66
*

*
mmHg 95% LOA -1.71 to 3.03); and in the increased lactate group (5.17 mmHg 95% LOA -1.63 to 11.97). The analysis showed a correlation between lactate level and the difference between TcCO2 and PaCO2 (r= 0.79, p< 0.001) and a negative correlation between mean blood pressure and the dif- ference between TcCO2 and PaCO2 (r= -0.54, p= 0.001). Multiple regression analysis results showed that lactate level was independently associated with increased differences between TcCO2 and PaCO2 (Beta= 0.875, p< 0.001)
*

**Conclusion:** *TcCO2 monitoring may not be reliable in patients with increased lactate levels. TcCO2 levels should be checked by ABG analysis in these patients.
*

**Key words:** *Transcutaneous carbondioxide; lactate; arterial blood gases; hypercapnia
*

**ÖZ**

**
Transkütanöz ve arteriyel ölçümler arasındaki laktat düzeyi ile karbondioksit basıncı ilişkisi
**

**Giriş:** *Alveoler ventilasyonun değerlendirilmesinde arteriyel kanın kısmi karbondioksit basıncı (PaCO2) kullanılır. Transkütanöz kar- bondioksit basıncı (TcCO2) izlemesi, arteriyel kan gazı analizine (ABG) non-invaziv (NİV) bir alternatif olarak geliştirilmiştir. Çalışmalar azalan doku perfüzyonunun karbondioksitin (CO2) artmasına yol açtığını göstermiştir. Perfüzyon anormallikleri olan has- talarda transkütanöz kapnometrinin kullanımı güvenilmez olabilir. Çalışmamızda hipoperfüzyonun belirteci olarak kabul edilen laktat düzeyi ile TcCO2-PaCO2 arasındaki ilişkiyi değerlendirmeyi amaçladık.
*

**Materyal ve Metod:** *Bu prospektif kohort çalışmada yoğun bakım ünitesinde Nisan 2019 ile Ocak 2020 arasında NİV alan hiperkap- nik solunum yetmezliği olan (PaCO2 ≥45 mmHg) yoğun bakım hastaları çalışmaya dahil edildi. Hastaların eş zamanlı ölçülen TcCO2 ve PaCO2 değerleri kaydedildi. Her eşleştirilmiş ölçüm normal laktat (<2 mmol/L) ve artmış laktat (≥2 mmol/L) olmak üzere iki gruba ayrıldı.
*

**Bulgular:** *Toplam 29 hastanın 116 eşleştirilmiş TcCO2 ve PaCO2 ölçümü kaydedildi. Bland-Altman analizi, tüm ölçümlerde TcCO2 ve PaCO2 arasındaki ortalama sapmayı ve %95 uyum limitlerini (LOA) gösterdi (1,75 mmHg %95 LOA -3,67 ile 7,17); normal laktat grubunda (0,66 mmHg %95 LOA -1,71 ile 3,03); ve laktat artışı olan grupta (5,17 mmHg %95 LOA -1,63 ile 11,97). Analiz, laktat düzeyi ile TcCO2 ve PaCO2 arasındaki fark (r= 0,79, p< 0,001) arasında pozitif bir korelasyon ve ortalama kan basıncı ile TcCO2 ve PaCO2 arasındaki fark (r= -0,54, p= 0,001) arasında negatif bir korelasyon gösterdi. Çoklu regresyon analizi sonuçları, laktat seviye- sinin bağımsız olarak TcCO2 ve PaCO2 arasındaki artan farklarla ilişkili olduğunu gösterdi (Beta= 0,875, p< 0,001).
*

**Sonuç:** *Laktat düzeyi yüksek olan hastalarda TcCO2 takibi güvenilir olmayabilir. Bu hastalarda ABG analizi ile TcCO2 düzeylerine bakılmalıdır.
*

**Anahtar kelimeler:** *Transkütan karbondioksit; laktat; arter kan gazı; hiperkapni
*

## INTRODUCTION

Partial carbon dioxide pressure (PaCO2) is used as an essential parameter in evaluating the adequacy of alveolar ventilation. Different pathophysiological conditions such as increased dead space, reduced respiratory drive, and increased work of breathing promote increased PaCO2 (1,2). Arterial blood gas (ABG) is considered the gold standard for assessing PaCO2 level. Patients with the conditions described above, especially those receiving non-invasive (NIV) or invasive mechanical ventilation support, need fre- quent ABG monitoring. However, it is a painful pro- cedure and carries a potential risk for bleeding, hematoma and thrombosis (3,4). These conditions have led to the search for non-invasive alternative methods for measuring PaCO2, and thus, the end- tidal the partial CO2 pressure (EtCO2) and the trans- cutaneous partial pressure of CO2 (TcCO2) have been developed (1). Transcutaneous carbon dioxide moni- toring was first described in the 1960s and has been continually improved in the following years (5). Good agreement between TcCO2 and PaCO2 has been shown in studies in children and adult popula- tions (6-10). On the contrary, several studies suggest that transcutaneous measurement of CO2 may not be reliable in patients with increased PaCO2 (11,12).

TcCO2 is dependent on the local and systemic perfu- sion of the patient. Particularly in the presence of circulatory failure, decoupling occurs between arte- rial PaCO2 and TcCO2, and an increase in TcCO2

occurs independently from arterial PaCO2. In accord- ance with this, it was demonstrated that altered tissue perfusion leads to elevated tissue CO2 and the differ- ence between TcCO2 and PaCO2 levels (4). Lactate is a widely used marker for the indirect assessment of tissue perfusion and/or hypoxia (4,13). In this paper, we hypothesized that transcutaneous capnometry may be unreliable in patients with tissue perfusion impairment/hypoxia, which can be represented by hyperlactatemia. To our knowledge, there are no studies focusing on the relationship of lactate level on measurements of TcCO2.

The purpose of this study was to evaluate the effect of lactate levels on the agreement of TcCO2 and PaCO2 in hypercapnic patients who receive NIV.

## MATERIALS and METHODS

**Study Population**

We conducted a prospective cohort study in critical care patients with hypercapnic respiratory failure (PaCO2 ≥45 mmHg) who received NIV between April 2019 and January 2020 in the intensive care unit of Ankara University Faculty of Medicine department of chest disease. Patients aged ≥18 years, patients receiving NIV as initial ventilation support due to hypercapnic respiratory failure, and those whose TcCO2 measurement could be recorded continuous- ly during the first 24 hours were enrolled in the study exclusion criteria were defined as follows: Requiring emergency intubation at critical care admission;

requiring invasive mechanical ventilation within the first 24 hours of NIV therapy; low systemic blood pressure (mean arterial pressure <60 mmHg); requir- ing vasopressor treatment during ICU stay; acute or chronic renal/hepatic failure, drug intoxication and the presence of a skin lesion that prevents placement of the transcutaneous capnometry probe; or the ina- bility of monitoring transcutaneous measurements for any reason. The presence of mechanisms such as multiple organ dysfunctions, sepsis, vasopressor use, barbiturates and propofol, which often cause an increase in lactate levels in different ways, were more common in patients who required invasive mechani- cal ventilation, so patients receiving invasive mechan- ical ventilation were not enrolled in the study to maintain the clarity of findings.

Data for the sample size was taken from a study by Endo et al. (14). Considering the four repeated meas- urements (0th, 1st, 4th and 24th hours) of TcCO2 and

arterial PaCO2 for each patient, with an effect size of

0.41 and with a setting correlation of 0.29 for repeat-

ed measures, we calculated that 28 patients were needed to reach the power of 85% with an alfa level of 5%. The G*POWER 3.1.9.6 program was used to calculate the sample size.

The study was approved by the Institutional Ethics Review Committee (05-305-18). Informed consent was obtained from all study patients.

## Study Protocol, Measurements and Data Collection

The demographic features, comorbid conditions, and baseline laboratory parameters, including complete blood count data and renal/liver function tests were recorded at the time of critical care admission. ABG

parameters including pH, PaCO2, PaO2, SaO2, HCO3 and lactate level, arterial blood pressure and body

temperature of the study patients were recorded at 0, 1, 4 and 24 hours of NIV application. All the ABG samples were analyzed within five minutes of blood collection using the ABL800 blood gas analyzers (Radiometer Medical ApS, Denmark). The TcCO2 levels were monitored using the TOSCA TCM4 (Radiometer Ltd., Crawley, United Kingdom). The transcutaneous electrode includes a heating piece, temperature sensor, and an H+ measuring electrode. Heating the skin provokes regional vasodilation and increased CO2 permeability. CO2 that diffuses through the membrane reacts with water to form carbonic acid, which then dissociates into hydrogen and bicarbonate ions. This results in a change in pH

that causes a potential difference between the two electrodes. Based on the linear relationship between pH and Log PCO2, TcCO2 is recorded. TcCO2 repre- sents a mixture of venous, capillary, and arterial CO2 pressures (4). According to the manufacturer’s instruc- tions, we performed the following steps for the trans- cutaneous measurements: A sensor fixation ring was placed on the previously cleaned upper anterior chest wall, and the conductive gel was dropped on the ring. Besides, sensor calibration was automati- cally done after each monitoring, and the membrane was changed every 14 days. TcCO2 values were

recorded simultaneously with the ABG sampling. Measurement method diagram is given in Figure 1.

The same clinicians performed all measurements to prevent interobserver reliability. Also, the clinicians and all staff are regularly trained and controlled. To ensure quality assurance of all measurements, the transcutaneous carbon dioxide monitor, arterial blood gases analyzers and the ventilator devices used for NIV have a program of periodic equipment main- tenance and ongoing monitoring established accord- ing to the manufacturer’s instructions.

As hyperlactatemia is defined as increased serum lactate level ≥2 mmol/L, all paired measurements are categorized into two different groups to evaluate the

differences of CO2 measurements as normal lactate group (<2 mmol/L) and increased lactate group (≥2

mmol/L) (15).

## Primary Endpoint

The primary endpoint was to assess the effect of lac- tate levels on the agreement in measurement between TcCO2 and PaCO2 in hypercapnic patients receiving NIV therapy.

## Statistical Analysis

Data were analyzed using SPSS 22.0 software (SPSS, Inc, Chicago, IL, United States of America). Continuous variables with normal distribution were presented as mean ± standard deviation (mean ± SD) and median [25th-75th percentiles, interquartile range (IQR)] for non-normal distributed variables. Kolmogorov-Smirnov test was used to analyze the distribution of variables and a Levene test to assess the equality of variances. The Bland-Altman method was performed to display bias (systematic error-mean the difference between TcCO2 and PaCO2) and precision (random error-standard deviation of mean difference) and were calculated. Mean difference

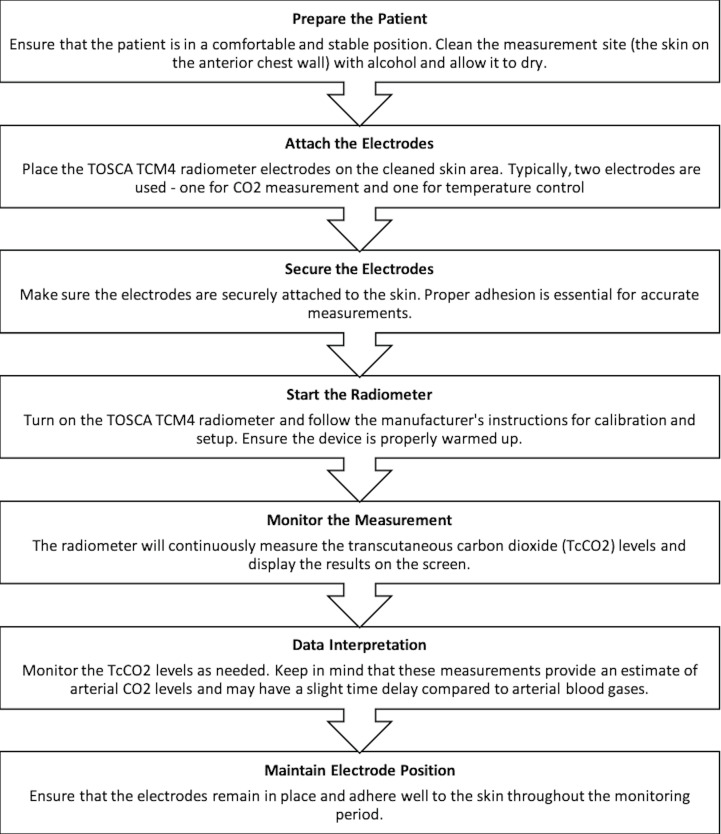

**Figure 1.** Measurement of transcutaneous carbon dioxide method diagram.

between TcCO2 and PaCO2 were given with 95% limits of agreement (± 1.96 SD). Repeated measures analysis of variance was used to determine changes of PaCO2, TcCO2 and differences of TcCO2 and PaCO2 over time. A Pearson’s correlation analysis was calculated to assess the strength of the

associations between any two variables. A multiple linear regression model was performed with enter method to identify independent predictors of the difference between TcCO2 and PaCO2. The model fit was assessed using appropriate residual and goodness-of-fit statistics. Tolerance and variance

inflation factor were used to determine the collinearity between independent variables. Statistical significance level was set as p< 0.05 for all tests.

## RESULTS

A total of 116 paired TcCO2 and PaCO2 measure- ments of 29 patients were enrolled for the study. Flow chart of the study patients is presented in Figure 2. Mean age of the study patients was 69.44 ± 10.62 years. The characteristics of the 29 study subjects are given in Table 1. The 116 paired mesaurements were categorized into increased lactate level (n= 28) and normal lactate level (n= 88).

The following clinical data were collected at the time of each TcCO2-PaCO2 measurement: Mean tempera- ture 36.54 ± 0.35 °C, oxygen saturations 90.84 ±

5.18 %, and mean arterial blood pressure 82.19 ±

13.27 mmHg.

Mean TcCO2 and PaCO2 levels were 58.30 ± 10.68 and 56.54 ± 10.86, respectively. Mean bias between TcCO2 and PaCO2 was 1.75 mmHg with 95% limits of agreement from -3.67 to 7.17 mmHg (Figure 3).

Paired measurements tested in 29 patients during 24 hours (analysed for 0, 1, 4 and 24 hours of NIV sup- port) did not show any significant change in TcCO2, PaCO2 and differences of TcCO2 and PaCO2 over the time (p= 0.09; p= 0.09; p= 0.64 respectively,

Figure 4).

When the measurements were grouped according to lactate levels, in the normal lactate level group (<2 mmol/L), the mean bias between TcCO2 and PaCO2 was 0.66 mmHg with 95% limits of agree- ment from -1.71 to 3.03 mmHg (Figure 5A), and TcCO2 levels showed high correlation with the PaCO2 levels (r= 0.99, p< 0.001, Figure 5B).

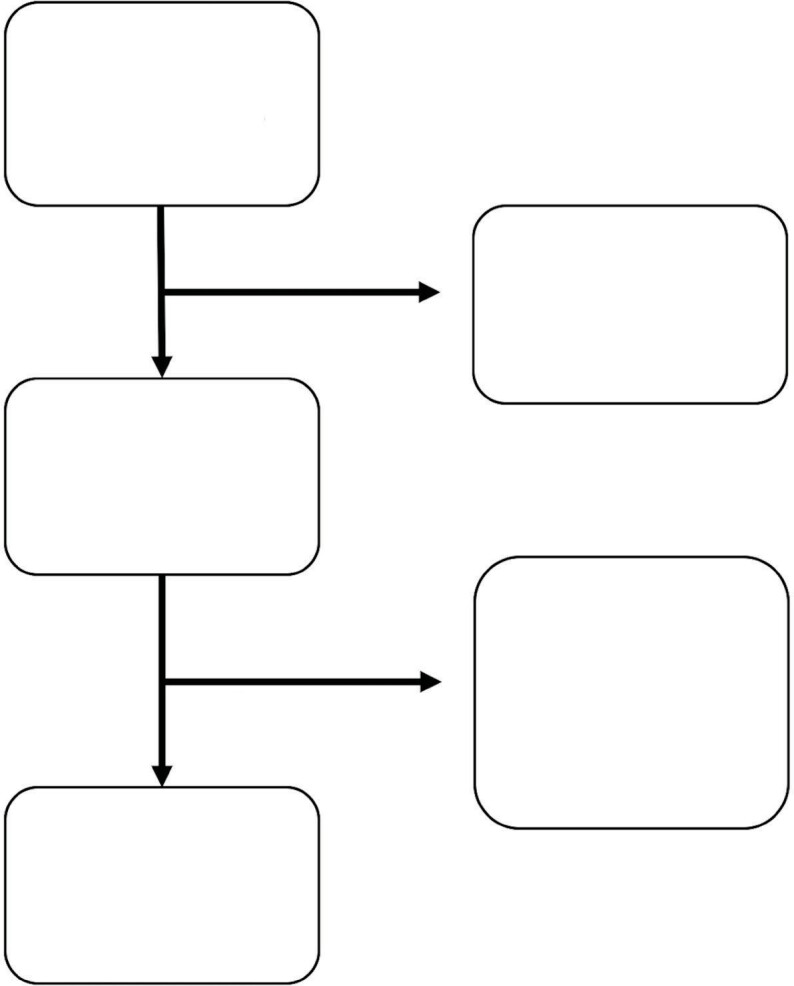

**Figure 2.** Flow diagram of the study.

**Table:** 

**Table 1.** General characteristics of the study patients	
**n= 29**	
**mean ± SD**	
**median (IQR25-75)**	
**n (%)**	
Age (year)	69.44 ± 10.62
Sex (male)	17 (58.6)
Reason for hypercapnic respiratory failure	
COPD exacerbation	18 (62)
Pulmonary edema	4 (13.8)
Pneumonia	3 (10.3)
Interstitial lung disease	2 (6.9)
Neuromuscular disease	2 (6.9)
Laboratory parameters	
Hb (g/dL)	11.81 ± 2.24
Leucocyte (109/L)	9.15 ± 2.68
Thrombocyte (109/L)	216.0 (172.5-295.0)
BUN (mg/dL)	29.0 (22.5-34.8)
Creatinin (mg/dL)	0.93 ± 0.31
ALT (U/L)	27.0 (17.5-37.0)
AST (U/L)	29 (16.5-39)
COPD: Chronic obstructive lung disease, Hb: Haemoglobin, BUN: Blood urea nitrogen, ALT: Alanine aminotransferase, AST: Aspartate aminotrans- ferase.

**Figure 3.** Bland-Altman plots for comparing TcCO2 with PaCO2 within the total study group. The X-axis represents the mean of TcCO2 with PaCO2 [(TcCO2+ PaCO2)/2] in mmHg and the Y-axis represents the difference between TcCO2 with PaCO2 (TcCO2-PaCO2) in mmHg. Red line shows the mean bias. Blue lines represent upper and lower limits of agreement at ± 1.96 SD.

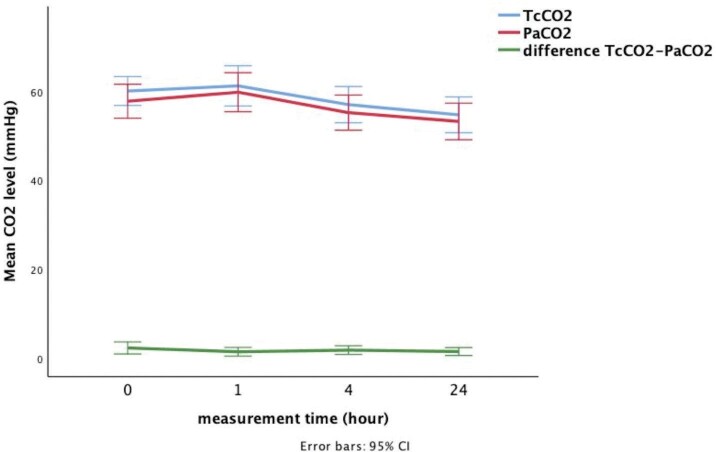

**Figure 4.** Graph represents the changes of TcCO2 (blue line), PaCO2 (red line) and difference between TcCO2 and PaCO2 (green line) over the 24 hours with the 95% CI.

**Figure 5.** Bland-Altman plots for comparing TcCO2 with PaCO2 within the normal lactate group. The X-axis represents the mean of TcCO2 with PaCO2 [(TcCO2+ PaCO2)/2] in mmHg and the Y-axis represents the difference between TcCO2 with PaCO2 (TcCO2- PaCO2) in mmHg. Red line shows the mean bias. Blue lines represent upper and lower limits of agreement at ±1.96 SD **(A)**. Association between TcCO2 and PaCO2 for normal lactate group **(B)**.

Besides this, within the high lactate group, mean bias between TcCO2 and PaCO2 was calculated as 5.17 mmHg with 95% limits of agreement from -1.63 to

11.97 mmHg (Figure 6A). TcCO2 levels still showed high correlation with the PaCO2 levels (r= 0.96, p< 0.001, Figure 6B).

Our findings showed a positive correlation of lactate level and with difference between TcCO2 and PaCO2 (r= 0.79, p< 0.001) (Figure 7A) and a negative cor- relation between mean blood pressure and the differ-

ence between TcCO2 and PaCO2 (r= -0.54, p= 0.001, Figure 7B). No other correlations were found with the difference between TcCO2 and PaCO2. After multiple regression analyses, the relation between lactate level and the difference between TcCO2 and PaCO2 remained, while the association of mean blood pressure became insignificant. Multiple regres- sion analysis of possible variables affecting the increase of difference between TcCO2 and PaCO2 values is given in Table 2.

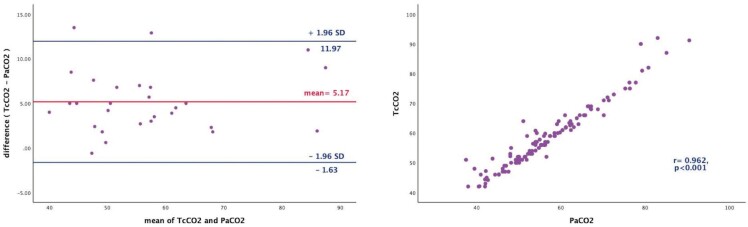

**Figure 6.** Bland-Altman plots for comparing TcCO2 with PaCO2 within increased lactate group. The X-axis represents the mean of TcCO2 with PaCO2 [(TcCO2+ PaCO2)/2] in mmHg and the Y-axis represents the difference between TcCO2 with PaCO2 (TcCO2- PaCO2) in mmHg. Red line shows the mean bias. Blue lines represent upper and lower limits of agreement at ±1.96 SD **(A)**. Association between TcCO2 and PaCO2 for increased lactate group **(B)**.

**Figure 7.** Association of difference between TcCO2 and PaCO2 with lactate level for all paired measurements **(A)**. Association of difference between TcCO2 and PaCO2 with mean arterial blood pressure for all paired measurements **(B)**.

**Table:** 

**Table 2.** Multiple linear regression analysis: Association between the variables and the difference between TcCO2 and PaCO2					
	**B**	**95% CI**	**Beta**	**t**	**p**
pH	2.599	-5.559-10.757	0.048	0.631	0.529
PaO2 (mmHg)	0.007	-0.13-0.27	0.042	0.701	0.485
PaCO2 (mmHg)	-0.003	-0.038-0.034	-0.010	-0.139	0.890
Mean blood pressure (mmHg)	0.019	-0.018-0.055	0.089	1.012	0.314
Lactate level (mmol/L)	3.080	2.446-3.713	0.875	9.632	**<0.001**
Temperature	0.110	-0.819-1.038	0.014	0.234	0.815
R^2^= 0.630, Adj R^2^= 0.610, F-value: 30.96, p< 0.001. PaO2= Arterial partial oxygen pressure, PaCO2= Arterial partial carbon dioxide oxygen pressure. B= Unstandardized co-efficients, Beta= Standardized co-efficients, CI= Confidence interval. p< 0.05 is considered as the significant level.					

## DISCUSSION

The findings of our study revealed a positive correlation between serum lactate level and the

difference of TcCO2 and PaCO2. Among all paired measurements, the mean bias between TcCO2 and PaCO2 was 1.75 mmHg with 95% limits of agreement from -3.67 to 7.17 mmHg. Besides that, the bias

between TcCO2 and PaCO2 increased as 5.17 mmHg with 95% limits of agreement from -1.63 to 11.97 mmHg, within the measurements with increased lactate (≥2 mmol/L). To the best of our knowledge, this is the first study targeting to investigate the effect of serum lactate level on transcutaneous CO2 measurement in critical care patients.

In clinical practice, PaCO2 is one of the main moni- toring markers, especially in intensive care units, and ABG analysis is accepted as the gold standard tech- nique for measuring it. In line with this, arterial blood sampling is the most extensive procedure for CO2 monitoring worldwide (1). Since arterial blood gas sampling is painful and carries risks such as thrombo- sis and hematoma, alternative methods are searched for CO2 monitoring (4). Arterial lines are sometimes placed to avoid repeated punctures, but it is also associated with significant risks, including infection and ischemia (16). Although the development of transcutaneous capnography for this purpose dates back a few decades, its usage is still quite limited, and data on its reliability in spesific patients group is insufficient.

Serum lactate level has been a widely used biomark- er for assessing hemodynamic conditions of the patients (13,17). The association between tissue hypoxia and elevated lactate level is known as the result of decreasing perfusion and reducing the com- ponents of systemic oxygen distribution due to low levels of haemoglobin, oxygen saturation, or cardiac output (13). Parallel to this, lactate level is used sur- rogate of tissue perfusion as it is influenced by mac- rocirculation, microcirculation (including arterioles, capillaries, and venules network), and mitochondrial

function. The cutaneous CO2 represents the CO2 value of the mixture of venous, capillary, and arterial

networks. The level of tissue CO2 is based on the production of CO2 by tissues, the wash-out capacity of CO2 from the tissue through enough perfusion, and arterial CO2 content (3). Previous studies have clearly shown that tissue CO2 is increased in shock states, which is closely related to tissue perfusion impairment (18-20).

Regarding TcCO2 measurements in circulatory alter- ations, a prospective observational study has sug- gested that TcCO2 monitoring could be used in patients with acutely developed hypotension (9). In a pig-model study by Endo et al., it has been reported the TcCO2-PaCO2 level markedly negatively corre- lated with cardiac output (CO) measured by both

pulmonary arterial catheterization (PAC) and the pulse index continuous cardiac output (PiCCO) (CO by PAC: r= -0.79 p< 0.001; CO by PiCCO: r= -0.74; p< 0.001) (14).

Moreover, several studies advocate the monitoring of TcCO2 to predict the status of hypoperfusion (4,14,21). The decoupling of TcCO2 and PaCO2 explains this condition in cases of circulatory failure in a famous study examining the kinetics of tissue CO2 during shock status (22). Notably, monitoring the difference between tissue and arterial PCO2 is more beneficial than the absolute value of TcPCO2 in detecting perfusion alterations (4). In our study group, mean arterial blood pressure was negatively correlated with the difference between TcCO2 and PaCO2. However, after multiple regression analyses, the association became insignificant. Nonetheless, the association between lactate level and increased TcCO2 and PaCO2 remained even after multiple analyses. We believe that lactate level may be a stronger indicator of tissue perfusion than arterial blood pressure since it is also affected by microcircu- latory dysfunciton.

Previous studies have suggested 7.5 mmHg as the clinically acceptable limit for the maximum mean bias between CO2 measurement methods (6,23,24). A study conducted in the emergency department by McVicar and Eager has reported a bias of 0.15 mmHg and limits of agreement of -6.6 to -6.9 mmHg (25). In another study, the bias has been found as 0.1 mmHg and limits of agreement of as -6 to 6.2 mmHg within a similar patient population (26). Mummary et al. have performed a study with patients requiring

acute medical settings and reported TcCO2 tended to be -0.16 kPa [~1.20 mmHg (95% CI ± 1.54 kPa

(~11.55 mmHg)] lower than PaCO2 (27). Another study examining the agreement of TcCO2 and PaCO2 during chronic obstructive pulmonary disease (COPD) exacerbation has found the bias for TcCO2 and PaCO2 to be 2.3 mmHg (-3.8 to 8.3 mmHg) (28). Our results of total measurements seem to concur reasonably with previous studies in terms of agree- ment of TcCO2 and PaCO2 with slight bias and acceptable limits of agreement for all measurements. In contrast, the mean TcCO2-PaCO2 difference (bias) increased from 0.66 mmHg in the normal lactate group to 5.17 mmHg in the increased lactate group. Furthermore, the limits of agreement got wider from normal lactate group to increased lactate group (from

-1.71 to 3.03 mmHg; to -1.63 to 11.97 mmHg).

Beyond that, an acceptable clinical range of agree- ment has been recommended by The American Association for Respiratory Care, between TcCO2 and PaCO2 ± 1.96 SD= ± 7.5 mmHg or narrower (29). Considering these data, we think transcutane- ous CO2 monitoring may not be reliable in cases with hyperlactatemia.

Our study had some limitations. It was carried out in a single centre. We used only one type of sensor device, and transcutaneous measurements were only per- formed on the anterior chest wall. Furthermore, the absence of measurements related to macrocirculation indicators, such as cardiac output and left ventricle ejection fraction, as well as the lack of data concern- ing microcirculatory status, including microcirculatory blood flow and postcapillary venous oxygen satura- tion, might have influenced the findings regarding the relation between lactate levels and TcCO2.

To conclude, TcCO2 monitoring may be a beneficial tool for estimating PaCO2 in critical care patients with hypercapnic respiratory failure receiving NIV. But, it may not be reliable in monitoring patients with increased lactate levels. For this reason, patients with known high lactate levels should be followed up with ABG as TcCO2 monitoring may provide doubtful results. In addition, TcCO2 measurements should be confirmed with ABG in cases with suspected hyperlactatemia in their follow-up. Further carefully designed studies taking into account different causes of hyperlactatemia and possible confounders are necessary to demonstrate the relationship between lactate levels and transcutaneous carbon dioxide measurements.

**Ethical Committee Approval:** This study was approved by the Ankara University Faculty of Medicine Clinical Research Ethics Committee (Decision no: 05-305-18, Date: 12.03.2018).

## CONFLICT of INTEREST

The authors declare that they have no conflict of interest.

## AUTHORSHIP CONTRIBUTIONS

Concept/Design: AGK, ŞNÖ, AK Analysis/Interpretation: AGK

Data acqusition: AGK, MÖ, ŞNÖ, SE, FA Writing: AGK, MÖ, SE, FA

Clinical Revision: All of authors Final Approval: AGK, AK

## REFERENCES

1 *
  Umeda A, Ishizaka M, Ikeda A, Miyagawa K, Mochida A, Takeda H, et al. Recent insights into the measurement of carbon dioxide concentrations for clinical practice in respiratory medicine. Sensors (Basel) 2021; 21(16): 5636. https://doi.org/10.3390/s21165636*
2 *
  Gürün Kaya A, Öz M, Akdemir Kalkan I, Gülten E, Çınar G, Azap A, et al. Is pulse oximeter a reliable tool for non-critically ill patients with COVID-19? Int J Clin Pract 2021; 75(12): e14983. https://doi.org/10.1111/ijcp.14983
  *
3 *
  Horvath CM, Brutsche MH, Baty F, Rudiger JJ. Transcutaneous versus blood carbon dioxide monitoring during acute non-invasive ventilation in the emergency department - a retrospective analysis. Swiss Med Wkly 2016; 146: w14373. https://doi.org/10.4414/smw.2016.14373*
4 *
  Mari A, Nougue H, Mateo J, Vallet B, Vallee F. Transcutaneous PCO2 monitoring in critically ill patients: Update and perspectives. J Thorac Dis 2019; 11(Suppl 11): S1558-S67. https://doi.org/10.21037/jtd.2019.04.64*
5. *
  Eberhard P. The design, use, and results of transcutaneous carbon dioxide analysis: Current and future directions. Anesth Analg 2007; 105(6 Suppl): S48-S52. https://doi.org/10.1213/01.ane.0000278642.16117.f8*
6. *
  Nicolini A, Ferrari MB. Evaluation of a transcutaneous carbon dioxide monitor in patients with acute respiratory failure. Ann Thorac Med 2011; 6(4): 217-20. https://doi.org/10.4103/1817-1737.84776*
7. *
  May A, Humston C, Rice J, Nemastil CJ, Salvator A, Tobias J. Non-invasive carbon dioxide monitoring in patients with cystic fibrosis during general anesthesia: End-tidal versus transcutaneous techniques. J Anesth 2020; 34(1): 66-71. https://doi.org/10.1007/s00540-019-02706-5*
8. *
  Spelten O, Fiedler F, Schier R, Wetsch WA, Hinkelbein J. Transcutaneous PTCCO2 measurement in combination with arterial blood gas analysis provides superior accuracy and reliability in ICU patients. J Clin Monit Comput 2017; 31(1): 153-8. https://doi.org/10.1007/s10877-015-9810-8*
9. *
  Kim JY, Yoon YH, Lee SW, Choi SH, Cho YD, Park SM. Accuracy of transcutaneous carbon dioxide monitoring in hypotensive patients. Emerg Med J 2014; 31(4): 323-6. https://doi.org/10.1136/emermed-2012-202228*
10. *
  Yumrukuz Senel M, Fırat H, Kurt EB. Evaluation of transcu- taneous carbon dioxide and saturation monitoring during fiberoptic bronchoscopy. Tuberk Toraks 2020; 68(4): 379- 87. https://doi.org/10.5578/tt.70356*
11. *
  Huttmann SE, Windisch W, Storre JH. Techniques for the measurement and monitoring of carbon dioxide in the blood. Ann Am Thorac Soc 2014; 11(4): 645-52. https://doi.org/10.1513/AnnalsATS.201311-387FR*
12. *
  Ruiz Y, Farrero E, Cordoba A, Gonzalez N, Dorca J, Prats E. Transcutaneous carbon dioxide monitoring in subjects with acute respiratory failure and severe hypercapnia. Respir Care 2016; 61(4): 428-33. https://doi.org/10.4187/respcare.04283*
13. *
  Bakker J, Nijsten MW, Jansen TC. Clinical use of lactate monitoring in critically ill patients. Ann Intensive Care 2013; 3(1): 12. https://doi.org/10.1186/2110-5820-3-12*
14. *
  Endo Y, Hirokawa T, Miyasho T, Takegawa R, Shinozaki K, Rolston DM, et al. Monitoring the tissue perfusion during hemorrhagic shock and resuscitation: Tissue-to-arterial carbon dioxide partial pressure gradient in a pig model. J Transl Med 2021; 19(1): 390. https://doi.org/10.1186/s12967-021-03060-5*
15. *
  Godinjak A, Jusufovic S, Rama A, Iglica A, Zvizdic F, Kukuljac A, et al. Hyperlactatemia and the importance of repeated lactate measurements in critically ill patients. Med Arch 2017; 71(6): 404-7. https://doi.org/10.5455/medarh.2017.71.404-407*
16. *
  Garg K, Howell BW, Saltzberg SS, Berland TL, Mussa FF, Maldonado TS, et al. Open surgical management of com- plications from indwelling radial artery catheters. J Vasc Surg 2013; 58(5): 1325-30. https://doi.org/10.1016/j.jvs.2013.05.011*
17. *
  Kushimoto S, Akaishi S, Sato T, Nomura R, Fujita M, Kudo D, et al. Lactate, a useful marker for disease mortality and severity but an unreliable marker of tissue hypoxia/ hypoperfusion in critically ill patients. Acute Med Surg 2016; 3(4): 293-7. https://doi.org/10.1002/ams2.207*
18. *
  Almac E, Siegemund M, Demirci C, İnce C. Microcirculatory recruitment maneuvers correct tissue CO2 abnormalities in sepsis. Minerva Anestesiol 2006; 72(6): 507-19.
  *
19. *
  Marik PE. Regional carbon dioxide monitoring to assess the adequacy of tissue perfusion. Curr Opin Crit Care 2005; 11(3): 245-51. https://doi.org/10.1097/01.ccx.0000158091.57172.f9*
20. *
  Waelgaard L, Dahl BM, Kvarstein G, Tonnessen TI. Tissue gas tensions and tissue metabolites for detection of organ hypoperfusion and ischemia. Acta Anaesthesiol Scand 2012; 56(2): 200-9. https://doi.org/10.1111/j.1399-6576.2011.02572.x*
21. *
  Barneck M, Papa L, Cozart A, Lentine K, Ladde J, Nguyen L, et al. The utility of transcutaneous carbon dioxide mea- surements in the emergency department: A prospective cohort study. J Am Coll Emerg Physicians Open 2021; 2(4): e12513. https://doi.org/10.1002/emp2.12513*
22. *
  Tremper KK, Shoemaker WC. Continuous CPR monitoring with transcutaneous oxygen and carbon dioxide sensors. Crit Care Med 1981; 9(5): 417-8. https://doi.org/10.1097/00003246-198105000-00033*
23. *
  van Oppen JD, Daniel PS, Sovani MP. What is the potential role of transcutaneous carbon dioxide in guiding acute non-invasive ventilation? Respir Care 2015; 60(4): 484-91. https://doi.org/10.4187/respcare.03335*
24. *
  Sarrazin F, Tessler MJ, Kardash K, McNamara E, Holcroft C. Blood gas measurements using the Bayer Rapid Point 405: Are we basing our decisions on accurate data? J Clin Monit Comput 2007; 21(4): 253-6. https://doi.org/10.1007/s10877-007-9082-z*
25. *
  McVicar J, Eager R. Validation study of a transcutaneous carbon dioxide monitor in patients in the emergency department. Emerg Med J 2009; 26(5): 344-6. https://doi.org/10.1136/emj.2007.055715*
26. *
  Gancel PE, Roupie E, Guittet L, Laplume S, Terzi N. Accuracy of a transcutaneous carbon dioxide pressure monitoring device in emergency room patients with acute respiratory failure. Intensive Care Med 2011; 37(2): 348- 51. https://doi.org/10.1007/s00134-010-2076-1*
27. *
  Mummery V, Rogers E, Padmanaban V, Matthew D, Woodcock T, Bloch S. Transcutaneous carbon dioxide measurement is not a reliable alternative to arterial blood gas sampling in the acute medical setting. Eur Respir J 2019; 53(4): 1801726. https://doi.org/10.1183/13993003.01726-2018*
28. *
  Sorensen KM, Leicht RV, Carlsson CJ, Elvekjaer M, Porsbjerg C, Aasvang EK, et al. Agreement between transcutaneous monitoring and arterial blood gases during COPD exacer- bation. Respir Care 2021; 66(10): 1560-6. https://doi.org/10.4187/respcare.08510*
29. *
  Restrepo RD, Hirst KR, Wittnebel L, Wettstein R. AARC clinical practice guideline: Transcutaneous monitoring of carbon dioxide and oxygen: 2012. Respir Care 2012; 57(11): 1955-62. https://doi.org/10.4187/resp-care.02011*

